# Poisonous Properties of Glycerine

**Published:** 1876-11

**Authors:** 


Poisonous Properties of Glycerine.—M. Dujardin Beaumetz, having noticed the tendency to employ glycerine internally, in the treatment of diabetes, for instance, and believing that it ought to be reserved for external use, was induced to investigate its effects on the economy when administered in large doses. Berthelot has shown that this substance is a triatomic alcohol, and it should consequently produce effects identical with those of alcohol. The following are the conclusions he has derived from his experiments:

1. When chemically pure glycerine is introduced under the skin of a dog in quantities of from 2 to $4\frac{1}{2}$ drachms for every $2\frac{1}{5}$ pounds of the weight of the body, it causes death within twenty-four hours.

2. The symptoms of acute glycerism resemble, within certain limits, those of acute alcoholism.

3. The necroscopic lesions of acute glycerism are analogous to those of alcoholism, which leads to the supposition that the toxic actions of the two substances are very nearly the same.

4. From a therapeutic standpoint, the introduction of large quantities of glycerine into the body is not devoid of danger.

It is necessary, then, to use care in prescribing glycerine for internal use, and not to lose sight of the fact, that, like alcohol, glycerine will prove a successful or an unsuccessful remedy according to its dose and mode of administration.—*La France Medicale*, August 2, 1876.

